# Temporal overlap of intraoperative hypotension and hypocapnia and postoperative outcomes after major open intracranial surgery: a time-weighted dual-hit cohort study

**DOI:** 10.3389/fmed.2026.1946283

**Published:** 2026-10-01

**Authors:** Xing Yan, Xuemin Zou, Ruowen Yang, Jinyun Wang, Lemei Zhu

**Affiliations:** 1 Hunan Key Laboratory of the Research and Development of Novel Pharmaceutical Preparations, School of Public Health, Changsha Medical University, Changsha, China; 2 Hunan Provincial University Key Laboratory of the Fundamental and Clinical Research on Neurodegenerative Diseases, Changsha Medical University, Changsha, China

**Keywords:** cerebral perfusion, end-tidal carbon dioxide, hypocapnia, intracranial surgery, intraoperative hypotension, perioperative outcomes, temporal overlap, time-weighted exposure

## Abstract

**Background:**

Intraoperative hypotension reduces cerebral perfusion pressure, whereas hypocapnia may reflect either ventilation-related cerebral vasoconstriction or reduced pulmonary perfusion during a low-flow state. We evaluated whether the time-weighted overlap of hypotension and hypocapnia was associated with adverse postoperative outcomes after major open intracranial surgery.

**Methods:**

We conducted a retrospective cohort study using INSPIRE version 1.4.2. Adults undergoing major open intracranial surgery were included if mean arterial pressure (MAP) and end-tidal carbon dioxide (EtCO_2_) had at least 80% synchronous coverage. Dual-hit cerebral hypoperfusion burden (DHCB) was defined as the proportion of synchronously valid monitoring time with simultaneous MAP <65 mmHg and EtCO_2_ < 30 mmHg. The primary outcome was a composite of in-hospital death, postoperative intensive care unit stay of at least 2 days, or prolonged postoperative hospitalization. Patient-clustered multivariable logistic regression simultaneously modeled hypotension-only, hypocapnia-only, and concurrent exposure burdens.

**Results:**

The final cohort included 2,286 operations in 2,092 patients. Concurrent hypotension and hypocapnia occurred in 708 operations (31.0%), and the composite outcome occurred after 676 operations (29.6%). Each 5-percentage-point increase in DHCB was associated with higher odds of the composite outcome (adjusted odds ratio, 1.19; 95% CI, 1.10–1.28; *P* < 0.001), whereas hypotension-only and hypocapnia-only burdens were not independently associated with the outcome. Adding DHCB improved model fit but only minimally increased apparent discrimination (C-statistic, 0.764 to 0.767). Results remained consistent across progressively adjusted and sensitivity analyses. Restricted cubic spline analysis showed an overall association (*P* < 0.001) but no significant evidence of nonlinearity (*P* for nonlinearity = 0.143). The standardized risk increased from 27.3% at DHCB = 0% to 45.6% at DHCB = 30%, although estimates at higher burdens were less precise.

**Conclusions:**

Greater temporal overlap between intraoperative hypotension and hypocapnia was associated with adverse short-term postoperative outcomes beyond the corresponding non-overlapping exposure burdens. DHCB should be interpreted as a hypothesis-generating marker of concurrent physiological exposure rather than evidence of two independent causal insults, and requires external validation with neurologically specific outcomes before clinical application.

## Introduction

Adequate cerebral perfusion during intracranial surgery depends on the interaction between cerebral perfusion pressure and cerebrovascular resistance. Intraoperative hypotension may reduce the pressure gradient driving cerebral blood flow, whereas ventilation-induced hypocapnia causes cerebral vasoconstriction and increases cerebrovascular resistance (1, 2). Although cerebral autoregulation can buffer moderate physiological fluctuations, this compensatory capacity may be impaired or shifted in patients with intracranial pathology (3). Simultaneous hypotension and low EtCO_2_ may therefore identify a clinically relevant period of physiological vulnerability. However, EtCO_2_ can also decrease when cardiac output and pulmonary blood flow fall, even without an increase in ventilation; temporal concurrence could therefore reflect a shared low-flow process rather than two independent physiological insults.

The interpretation of hypocapnia in neurosurgery is complex. Controlled hyperventilation may be deliberately requested to reduce cerebral blood volume, intracranial pressure, and brain bulk and thereby improve surgical exposure (4, 5). Such purposeful, brief hyperventilation is clinically distinct from inadvertent hypocapnia or hypocapnia that persists after the surgical indication has resolved. Patients requiring hyperventilation for a tense or swollen brain may also have more severe intracranial pathology, greater operative complexity, and a higher baseline risk of adverse outcomes. Because the INSPIRE data do not record the indication, timing of the surgical request, or brain-relaxation assessment, these scenarios could not be separated. Accordingly, low EtCO_2_ should not be interpreted uniformly as a harmful exposure, and confounding by indication remains possible.

Previous perioperative studies have generally evaluated hypotension and carbon dioxide abnormalities separately and have rarely quantified their temporal concurrence (6–8). Conventional presence-vs.-absence or four-group classifications also ignore exposure duration and cannot distinguish sequential abnormalities from true simultaneous exposure. Thus, patients with similar cumulative durations of hypotension and hypocapnia may experience markedly different physiological exposure depending on the extent to which the two occur at the same time. Quantifying this temporal overlap may therefore capture clinically relevant information beyond the separate burdens of hypotension and hypocapnia.

We therefore defined a time-weighted dual-hit cerebral hypoperfusion burden (DHCB) as the proportion of valid intraoperative monitoring time during which mean arterial pressure (MAP) was <65 mmHg and end-tidal carbon dioxide (EtCO_2_) was <30 mmHg simultaneously. The term dual hit was used as an operational label for temporal overlap and did not presume two independent causal pathways, biological synergy, or a specific mechanism for the low EtCO_2_ value. Using the INSPIRE perioperative database, we evaluated whether greater DHCB was associated with adverse short-term postoperative outcomes after major open intracranial surgery after accounting for the non-overlapping burdens of hypotension and hypocapnia (9). We additionally examined whether temporal overlap provided incremental information beyond these individual exposure burdens, evaluated potential nonlinearity in the association, and estimated standardized absolute risks across clinically interpretable levels of DHCB.

## Methods

### Data source and study design

We conducted a retrospective observational cohort study using version 1.4.2 of the INSPIRE perioperative database, a de-identified single-center dataset containing linked demographic characteristics, operative and anesthetic records, time-stamped physiological measurements, laboratory results, diagnoses, medications, intensive care data, hospital length of stay, and mortality (9). The analysis was exploratory and hypothesis-generating and was reported in accordance with the principles of the STROBE statement and RECORD extension (10, 11).

### Study population

We included patients aged ≥18 years who underwent general anesthesia lasting ≥60 min for a major open intracranial procedure. High-specificity ICD-10-PCS prefixes were used to identify open brain tissue excision, open intracranial drainage, and open intracranial arterial occlusion or restriction. Spine procedures, cerebrospinal fluid shunt or external ventricular drainage procedures, stereotactic minor procedures, transsphenoidal procedures, and non-open vascular interventions were excluded. MAP and EtCO_2_ were required to have valid synchronous measurements during at least 80% of the reconstructed anesthesia period. Multiple eligible operations from the same patient were retained in the primary analysis because both the intraoperative exposures and postoperative outcomes were operation-specific. Retaining all eligible operations avoided the arbitrary exclusion of subsequent qualifying procedures and preserved the available information and precision. The resulting within-patient correlation was addressed using patient-clustered robust covariance estimation, and a sensitivity analysis restricted to the first eligible operation per patient was performed to assess the robustness of this approach.

### Exposure reconstruction

The anesthesia interval was reconstructed on a regular 5 min grid because the publicly available INSPIRE dataset provides intraoperative vital signs at a maximum temporal resolution of 5 min, with measurements recorded at shorter intervals aggregated to median values within each 5 min interval. Therefore, measurements at a shorter temporal resolution were not available in the public dataset. At each grid point, the nearest MAP and EtCO_2_ measurements within ±2.5 min were matched. Invasive MAP was preferentially used when available, with noninvasive MAP used otherwise. Values outside prespecified physiological ranges of 20–200 mmHg for MAP and 10–80 mmHg for EtCO_2_ were excluded. Only grid points with simultaneously valid MAP and EtCO_2_ measurements contributed to exposure calculations.

The primary exposure, dual-hit cerebral hypoperfusion burden (DHCB), was defined as the proportion of synchronously valid monitoring time during which MAP was <65 mmHg and EtCO_2_ was <30 mmHg simultaneously (6, 12). The EtCO_2_ threshold of 30 mmHg was prespecified to identify more pronounced hypocapnia rather than mild reductions commonly observed during general anesthesia. Effects were reported per 5-percentage-point increase in DHCB. To distinguish temporal concurrence from separate exposure burdens, valid monitoring time was partitioned into mutually exclusive states: hypotension only (MAP <65 mmHg and EtCO_2_ ≥ 30 mmHg), hypocapnia only (MAP ≥65 mmHg and EtCO_2_ < 30 mmHg), and concurrent hypotension and hypocapnia (MAP <65 mmHg and EtCO_2_ < 30 mmHg). The corresponding proportions of valid monitoring time were entered simultaneously in the core regression model.

### Outcomes

The exploratory primary outcome was a composite of in-hospital death, postoperative ICU stay ≥2 days, or postoperative hospital length of stay at or above the 75th percentile of the analytic cohort (9.62 days). Secondary outcomes were the individual components of the composite. ICU admission alone was not used because routine postoperative ICU pathways after intracranial surgery may reflect institutional practice rather than postoperative morbidity (13, 14).

### Covariates

The core clinical model included age, sex, body mass index, ASA physical status, emergency status, anesthesia duration, procedure group, hypotension-only burden, and hypocapnia-only burden. An extended preoperative model additionally included the most recent hemoglobin, creatinine, albumin, sodium, glucose, platelet count, and white blood cell count measured within 30 days before anesthesia, together with diagnosis groups for hypertension, diabetes mellitus, chronic kidney disease, ischemic heart disease, cerebrovascular disease, and malignancy. Missing continuous covariates were imputed using cohort medians with corresponding missingness indicators.

Estimated blood loss, transfusion, vasopressor administration, and ventilation-related variables could represent broader intraoperative severity, clinical responses, or post-exposure mediating processes and were therefore excluded from the primary model. These variables were incorporated only in a separate intraoperative-context sensitivity model, which included estimated blood loss, transfusion, vasopressor use, median minute ventilation, tidal volume, positive end-expiratory pressure, peak inspiratory pressure, and inspired oxygen fraction. Minute ventilation measurements were available for 1,790 operations (78.3%). Cardiac index and stroke volume index were recorded in only 313 (13.7%) and 244 (10.7%) operations, respectively, reflecting selective advanced hemodynamic monitoring; they were therefore considered too sparse for reliable multivariable adjustment. Patient position, intracranial pressure, baseline neurological function, and the indication for deliberate hyperventilation could not be reliably reconstructed.

### Statistical analysis

Operations were the units of analysis. Because patients could contribute more than one eligible operation, logistic regression models used the patient identifier as the clustering unit and a finite-sample-corrected cluster-robust sandwich covariance estimator. Adjusted odds ratios (aORs) with 95% confidence intervals (CIs) were reported per 5-percentage-point increase in DHCB. In the core model, hypotension-only burden, hypocapnia-only burden, and DHCB were entered simultaneously with the prespecified clinical covariates. The same core adjustment framework was used for analyses of the individual components of the composite outcome.

To evaluate whether temporal overlap contributed information beyond the non-overlapping exposure burdens, we compared a reduced model containing the clinical covariates, hypotension-only burden, and hypocapnia-only burden with a full model additionally containing DHCB. The additional DHCB term was evaluated using a patient-clustered robust Wald test. Relative model fit was described using the Akaike information criterion (AIC) and Bayesian information criterion (BIC), and apparent discrimination was summarized using the C-statistic (AUC) and the change in AUC after addition of DHCB. Because no resampling-based internal validation was performed, AIC, BIC, and discrimination estimates were treated as descriptive in-sample measures rather than validated predictive performance.

The stability of the DHCB association was further examined using progressive adjustment. The core clinical model was followed by an extended preoperative model incorporating laboratory measurements and diagnosis groups and, separately, an intraoperative-context sensitivity model incorporating blood loss, transfusion, vasopressor use, median minute ventilation, tidal volume, positive end-expiratory pressure, peak inspiratory pressure, and inspired oxygen fraction. The latter was treated as a sensitivity analysis rather than as a principal causal model because some intraoperative variables could occur after exposure onset and act as mediators or colliders. Cardiac index and stroke volume index were not included because their limited, nonrandom availability would have produced a small and highly selected complete-case subset.

Potential nonlinearity was assessed using restricted cubic splines within the core adjustment framework (15). Because DHCB was zero in a substantial proportion of operations, four knots were placed at the 5th, 35th, 65th, and 95th percentiles among DHCB-positive operations, corresponding to 1.2%, 2.6%, 6.2%, and 27.3%, with DHCB = 0% as the reference. Patient-clustered robust covariance estimates were used for overall and nonlinear Wald tests.

Marginal standardization was used to estimate adjusted population-averaged risks of the composite outcome at prespecified DHCB values of 0%, 5%, 10%, 20%, and 30% (16). For each value, DHCB was fixed at the specified level while the observed distributions of other covariates were retained, and predicted probabilities were averaged across the analytic cohort. Absolute risk differences were calculated relative to DHCB = 0%, with 95% CIs derived using the delta method and the patient-clustered robust covariance matrix.

Prespecified sensitivity analyses restricted the cohort to elective operations, open brain tissue excision procedures, the first eligible operation for each patient, and operations with ≥80% synchronous invasive-MAP coverage. Alternative concurrent-exposure definitions of MAP <70 mmHg with EtCO_2_ < 30 mmHg and MAP <65 mmHg with EtCO_2_ < 32 mmHg were also examined; the concurrent and corresponding non-overlapping burdens were reconstructed under each alternative threshold using the same time-grid approach.

All statistical analyses were performed using R version 4.5.1. All tests were two-sided. Given the exploratory and hypothesis-generating nature of the study, no adjustment for multiple comparisons was applied, and secondary, nonlinear, model-comparison, and sensitivity analyses were interpreted as exploratory.

## Results

### Study population

Of 2,353 candidate operations, 2,286 (97.2%) met the prespecified requirement of at least 80% synchronous MAP–EtCO_2_ coverage and were included in the final analytic cohort, representing 2,092 unique patients. Among these patients, 166 (7.9%) underwent more than one eligible operation. The cohort included 1,573 open brain tissue excision procedures (68.8%), 465 open intracranial vascular procedures (20.3%), and 248 open intracranial drainage procedures (10.8%). The study-selection process is presented in Figure 1, and demographic, procedural, preoperative, and intraoperative characteristics are summarized in Table 1.

**Figure 1:**
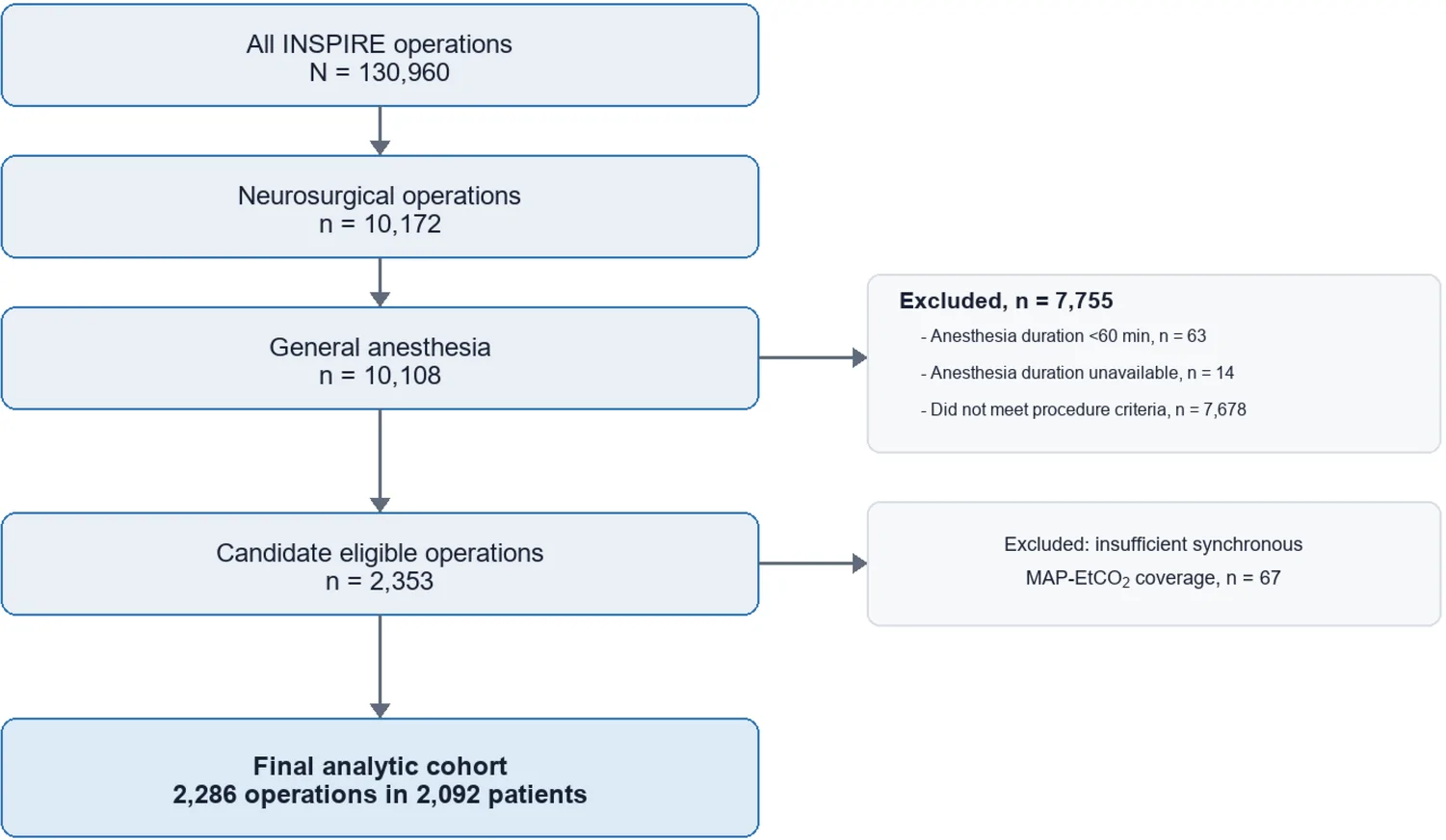
Study cohort construction.

**Table 1:** Characteristics of the analytic cohort.

Characteristic	Overall cohort
Cohort size	
Operations	2,286
Unique patients	2,092
Patients with >1 eligible operation	166 (7.9%)
Demographic and clinical characteristics	
Age, years	55.0 [45.0–65.0]
Female sex	1,299 (56.8%)
BMI, kg/m^2^	23.4 [21.5–26.0]
BMI, missing	53 (2.3%)
ASA physical status I–II	1,902 (83.2%)
ASA physical status III	269 (11.8%)
ASA physical status IV–V	28 (1.2%)
ASA physical status, missing	87 (3.8%)
Emergency surgery	238 (10.4%)
Procedure characteristics	
Open brain tissue excision	1,573 (68.8%)
Open intracranial vascular surgery	465 (20.3%)
Open intracranial drainage	248 (10.8%)
Anesthesia duration, min	270 [160–370]
Preoperatively recorded diagnosis groups	
Hypertension	143 (6.3%)
Diabetes mellitus	111 (4.9%)
Chronic kidney disease	22 (1.0%)
Ischemic heart disease	59 (2.6%)
Cerebrovascular disease	256 (11.2%)
Malignancy	742 (32.5%)
Preoperative laboratory measurements	
Hemoglobin, g/dL	13.2 [12.5–14.2]
Creatinine, mg/dL	0.74 [0.65–0.88]
Albumin, g/dL	4.1 [3.9–4.4]
Sodium, mmol/L	140 [139–141]
Glucose, mg/dL	120 [101–138]
Platelet count, ×10^9^/L	233 [200–273]
White blood cell count, ×10^9^/L	6.67 [5.58–8.90]
Intraoperative exposure characteristics	
Hypotension duration, min	15 [5–55]
Hypocapnia duration, min	10 [5–55]
Concurrent hypotension–hypocapnia duration, min	0 [0–5]
DHCB, % of synchronously valid monitoring time	0.0 [0.0–1.7]
Any concurrent hypotension–hypocapnia exposure	708 (31.0%)

Continuous variables are median [IQR]; categorical variables are *n* (%). Intraoperative exposures were reconstructed on a half-open regular 5-minute grid, with MAP and EtCO_2_ aligned separately; only operations with at least 80% synchronous valid coverage were included. Laboratory values were the closest available measurements within 30 days before anesthesia and were summarized using available observations. Missing values were: BMI, 53 (2.3%); ASA physical status, 87 (3.8%); hemoglobin, 140 (6.1%); creatinine, 77 (3.4%); albumin, 212 (9.3%); sodium, 53 (2.3%); glucose, 327 (14.3%); platelet count, 145 (6.3%); and white blood cell count, 143 (6.3%). Diagnosis groups were derived from codes recorded before anesthesia and may underestimate chronic disease prevalence. BMI, body mass index; ASA, American Society of Anesthesiologists; MAP, mean arterial pressure; EtCO_2_, end-tidal carbon dioxide; DHCB, dual-hit cerebral hypoperfusion burden.

### Intraoperative exposure patterns

The median cumulative duration of hypotension was 15 min (IQR, 5–55), the median duration of hypocapnia was 10 min (IQR, 5–55), and the median duration of concurrent hypotension and hypocapnia was 0 min (IQR, 0–5). The median DHCB was 0.0% of synchronously valid monitoring time (IQR, 0.0%–1.7%). DHCB was zero in 1,578 operations (69.0%), whereas 708 operations (31.0%) had at least one 5 min interval of concurrent MAP <65 mmHg and EtCO_2_ < 30 mmHg.

At the operation level, 102 operations (4.5%) had neither threshold exposure, 406 (17.8%) had hypotension only, 373 (16.3%) had hypocapnia only, 697 (30.5%) experienced both abnormalities without temporal overlap, and 708 (31.0%) experienced concurrent hypotension and hypocapnia. Compared with DHCB-negative operations, DHCB-positive operations involved older patients (median, 60 vs. 55 years), lower BMI (22.9 vs. 23.9 kg/m^2^), a higher proportion of open brain tissue excision (78.1% vs. 64.6%), a lower proportion of open intracranial vascular surgery (11.7% vs. 24.2%), and longer anesthesia duration (median, 305 vs. 255 min). Emergency surgery was similarly frequent (11.2% vs. 10.1%). The composite outcome occurred in 39.3% of DHCB-positive and 25.2% of DHCB-negative operations. These unadjusted descriptive differences, including the greater representation of brain tissue excision and longer procedures in the DHCB-positive group, are compatible with greater case complexity and potential confounding by indication. Full characteristics are reported in Supplementary Table S1; the operation-level exposure patterns and distribution of concurrent exposure duration are shown in Figure 2.

**Figure 2:**
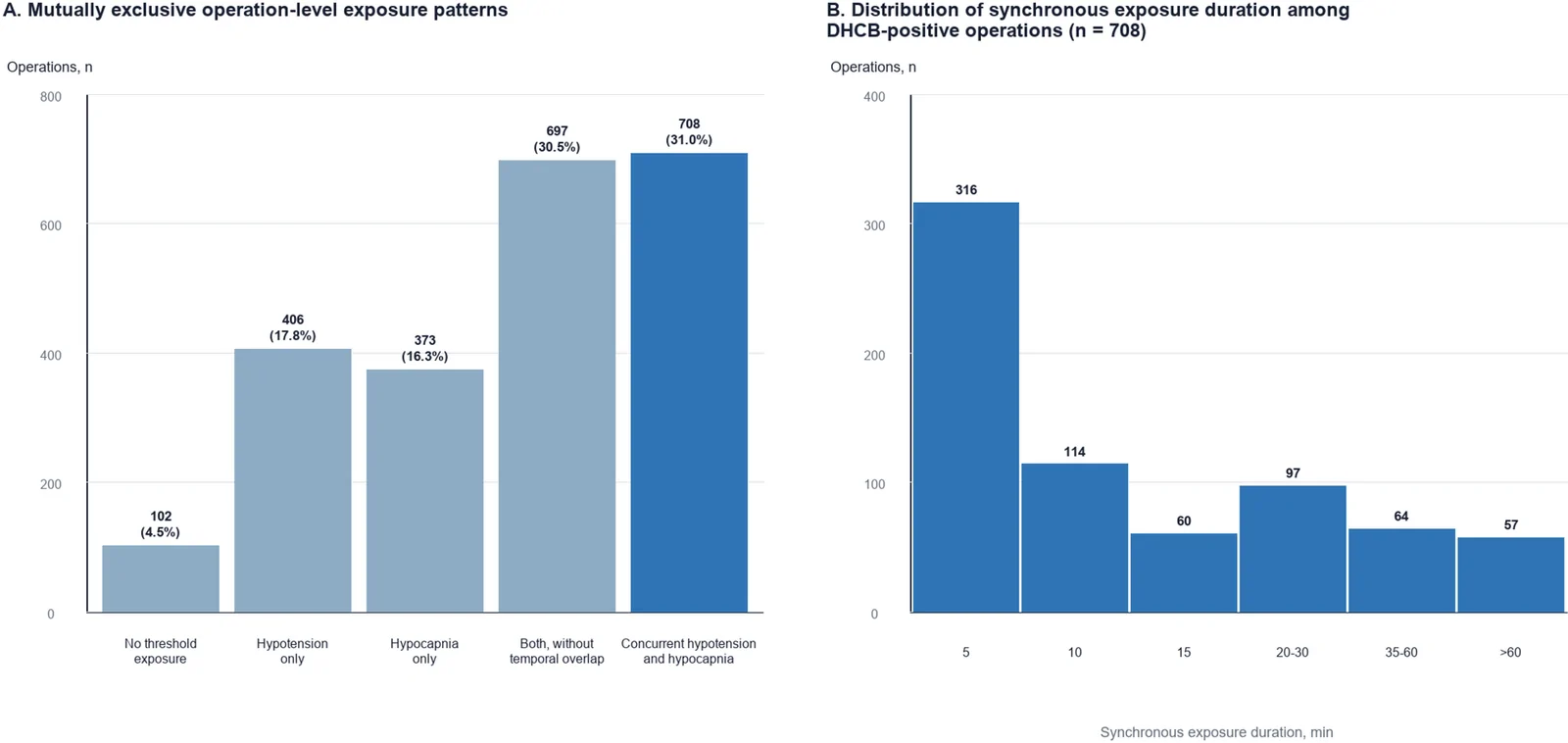
**(A)** Mutually exclusive operation-level exposure patterns. **(B)** Distribution of synchronous exposure duration among operations with concurrent hypotension and hypocapnia (*n* = 708).

### Association of DHCB with the composite adverse outcome

The composite adverse outcome occurred after 676 operations (29.6%). When hypotension-only, hypocapnia-only, and concurrent exposure burdens were entered simultaneously into the core clinical model, each 5-percentage-point increase in DHCB was associated with greater odds of the composite outcome (aOR, 1.19; 95% CI, 1.10–1.28; *P* < 0.001). In contrast, neither hypotension-only burden (aOR, 1.00; 95% CI, 0.96–1.03; *P* = 0.805) nor hypocapnia-only burden (aOR, 1.02; 95% CI, 0.99–1.04; *P* = 0.140) was independently associated with the outcome in the same model (Table 2).

**Table 2:** Patient-clustered associations of mutually exclusive intraoperative exposure burdens with the composite adverse outcome.

Exposure-state burden	aOR per 5-percentage-point increase	95% CI	*P* value
Hypotension-only burden	1.00	0.96–1.03	0.805
Hypocapnia-only burden	1.02	0.99–1.04	0.140
Concurrent hypotension-hypocapnia burden (DHCB)	1.19	1.10–1.28	<0.001

All three exposure burdens were entered simultaneously with age, sex, BMI, BMI missingness, ASA physical status categories, ASA missingness, emergency status, anesthesia duration, and procedure category. Effect estimates are per 5-percentage-point increase in the corresponding burden. Standard errors were clustered at the patient level using a finite-sample-corrected sandwich covariance estimator.

Adding DHCB improved model fit: the AIC decreased from 2,336.86 to 2,321.53 and the BIC decreased from 2,417.15 to 2,407.55. The patient-clustered robust Wald test for the additional DHCB term was significant (*χ*^2^ = 19.36; *P* < 0.001). However, the apparent C-statistic increased only from 0.764 to 0.767 (*Δ*AUC = 0.003), indicating little improvement in in-sample discrimination (Supplementary Table S2).

The association remained consistent across progressively adjusted analyses. The aOR per 5-percentage-point increase in DHCB was 1.19 (95% CI, 1.10–1.28; *P* < 0.001) in the core model, 1.17 (95% CI, 1.08–1.26; *P* < 0.001) after additional adjustment for preoperative laboratory measurements and diagnosis groups, and 1.15 (95% CI, 1.06–1.25; *P* < 0.001) in the intraoperative-context sensitivity model, which included median minute ventilation and other ventilation-related variables together with blood loss, transfusion, and vasopressor use. Restricting the analysis to the first eligible operation per patient produced a similar estimate (aOR, 1.20; 95% CI, 1.11–1.29; *P* < 0.001) (Figure 3 and Supplementary Table S3).

**Figure 3:**
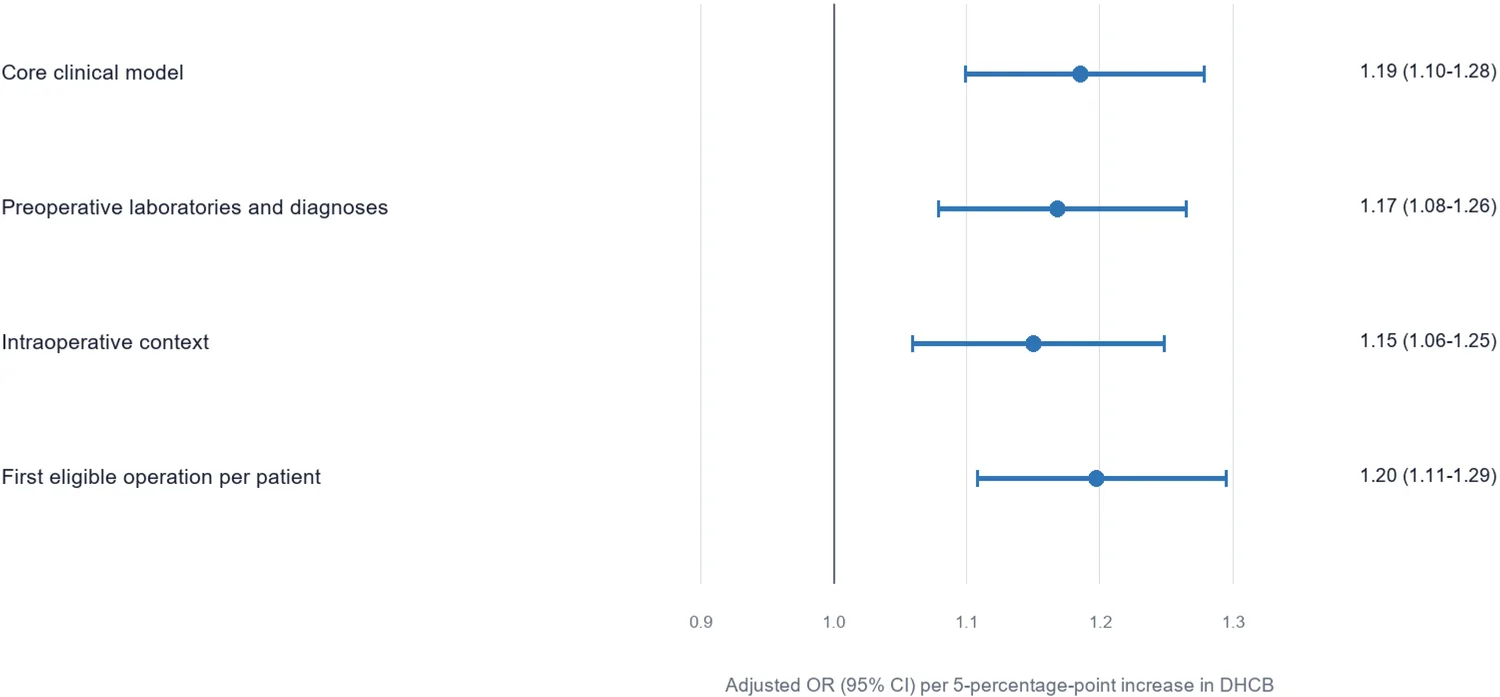
Association between DHCB and the composite adverse outcome across adjustment strategies.

### Individual outcome components

Prolonged postoperative hospitalization occurred after 572 operations, an ICU stay of at least 2 days occurred after 276 operations, and 53 patients died during hospitalization. For each 5-percentage-point increase in DHCB, the aOR was 1.10 (95% CI, 1.01–1.20; *P* = 0.029) for prolonged postoperative hospitalization, 1.15 (95% CI, 1.05–1.25; *P* = 0.003) for an ICU stay of at least 2 days, and 1.22 (95% CI, 1.07–1.39; *P* = 0.003) for in-hospital death (Table 3).

**Table 3:** Patient-clustered associations of DHCB with the composite adverse outcome and its individual components.

Outcome	Events	aOR	95% CI	*P* value
Composite adverse outcome	676	1.19	1.10–1.28	<0.001
Prolonged postoperative hospitalization	572	1.10	1.01–1.20	0.029
ICU stay ≥2 days	276	1.15	1.05–1.25	0.003
In-hospital death	53	1.22	1.07–1.39	0.003

Effect estimates are reported per 5-percentage-point increase in DHCB using the core clinical model with patient-level cluster-robust standard errors. Prolonged postoperative hospitalization was defined from the end of surgery to hospital discharge. Component analyses are exploratory.

### Continuous association and standardized absolute risks

Restricted cubic spline analysis demonstrated an overall association between DHCB and the composite adverse outcome (*P* < 0.001), but there was no statistically significant evidence of departure from linearity (*P* for nonlinearity = 0.143). Confidence intervals widened at higher DHCB values, where observations were less frequent (Figure 4).

**Figure 4:**
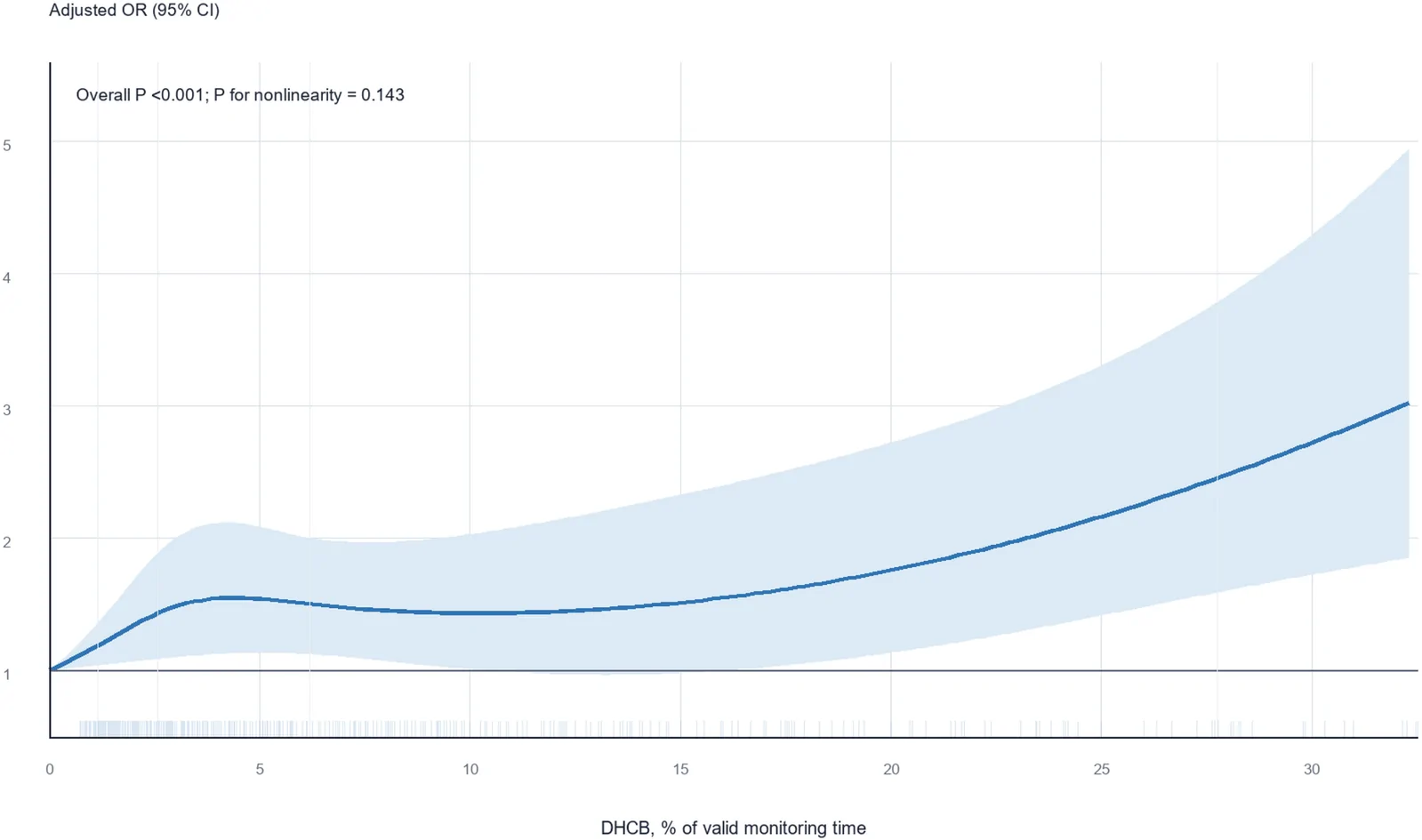
Restricted cubic spline association between DHCB and the composite adverse outcome.

The marginally standardized risks were 27.3%, 34.7%, 33.4%, 37.1%, and 45.6% when DHCB was set to 0%, 5%, 10%, 20%, and 30%, respectively. Compared with DHCB = 0%, the corresponding absolute risk differences were +7.4 percentage points (95% CI, 2.1–12.8), +6.1 percentage points (95% CI, −0.02 to 12.3), +9.9 percentage points (95% CI, 1.7–18.0), and +18.4 percentage points (95% CI, 9.4–27.4), respectively (Figure 5 and Supplementary Table S4).

**Figure 5:**
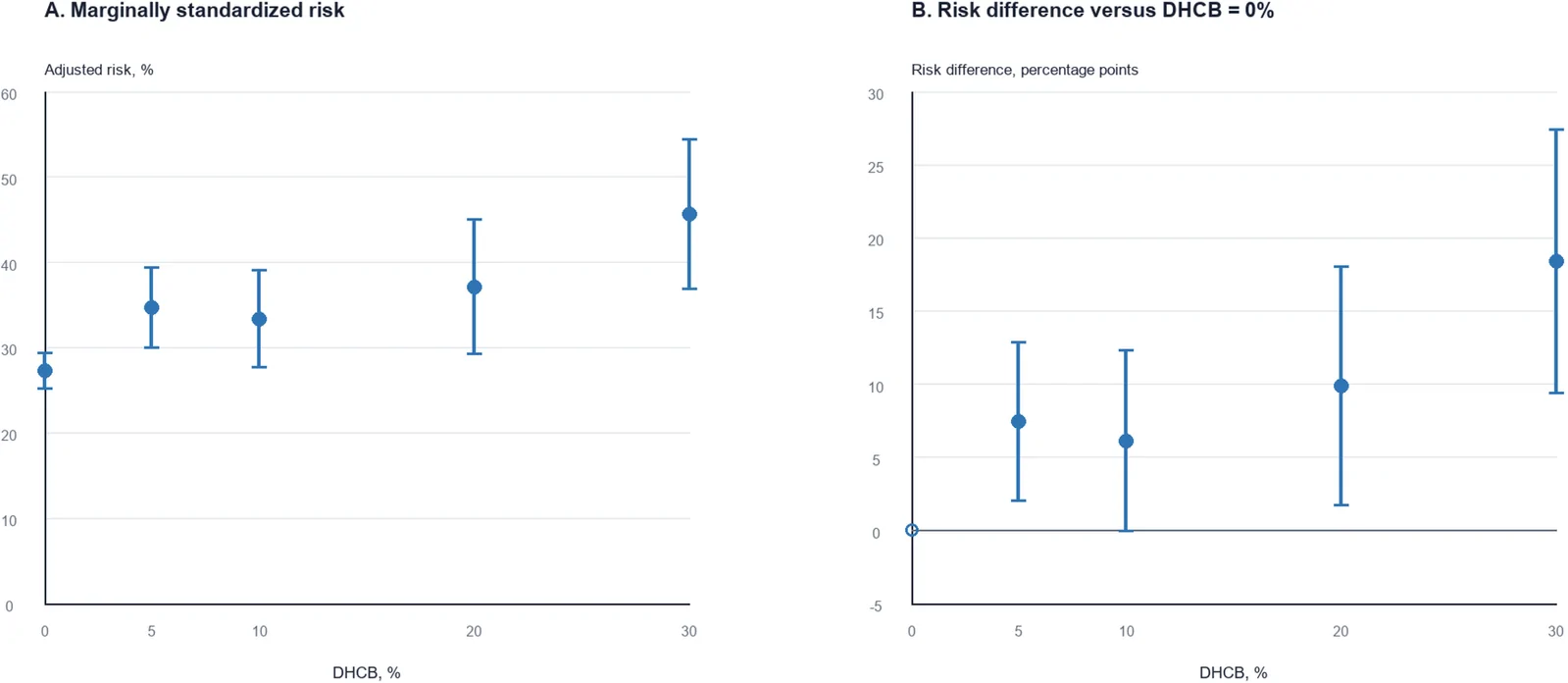
**(A)** Marginally standardized risk across selected DHCB levels. **(B)** Absolute risk difference relative to DHCB = 0% across selected DHCB levels.

### Sensitivity analyses

The association remained consistent in analyses restricted to elective operations (aOR, 1.19; 95% CI, 1.10–1.29), open brain tissue excision procedures (aOR, 1.19; 95% CI, 1.09–1.29), the first eligible operation per patient (aOR, 1.20; 95% CI, 1.11–1.29), and operations with at least 80% synchronous invasive-MAP coverage (aOR, 1.16; 95% CI, 1.08–1.25). The association also remained present using MAP <70 mmHg with EtCO_2_ < 30 mmHg (aOR, 1.12; 95% CI, 1.07–1.18; *P* < 0.001) and was weaker using MAP <65 mmHg with EtCO_2_ < 32 mmHg (aOR, 1.04; 95% CI, 1.01–1.08; *P* = 0.023) (Supplementary Table S5).

## Discussion

In this retrospective cohort of adults undergoing major open intracranial surgery, concurrent intraoperative hypotension and low EtCO_2_ was not uncommon, with nearly one third of operations experiencing at least one episode of simultaneous MAP <65 mmHg and EtCO_2_ < 30 mmHg. A greater dual-hit cerebral hypoperfusion burden (DHCB), used here as an operational measure of temporal overlap, was associated with higher odds of a composite short-term adverse postoperative outcome after accounting for the non-overlapping burdens of hypotension and hypocapnia and other prespecified clinical covariates. This association remained broadly consistent after additional adjustment for preoperative laboratory and diagnostic factors, in an intraoperative-context sensitivity model that included minute ventilation and other ventilation-related measures, and when the analysis was restricted to the first eligible operation per patient. Associations were also directionally consistent across the individual components of the composite outcome.

The principal contribution of this study is the explicit incorporation of temporal overlap into the characterization of intraoperative physiological exposure. Conventional analyses that evaluate hypotension and hypocapnia separately may fail to distinguish sequential abnormalities from truly concurrent exposure. In the present study, hypotension-only burden, hypocapnia-only burden, and DHCB were modeled simultaneously. Under this framework, DHCB remained associated with the composite outcome, whereas the two non-overlapping burdens were not independently associated in the same model. Adding DHCB to a model already containing the two non-overlapping exposure burdens improved model fit, as indicated by the patient-clustered robust Wald test and lower AIC and BIC values. These findings suggest that temporal concurrence contains statistical information not fully captured by the separate cumulative burdens alone. However, the increase in apparent discrimination was small, with the C-statistic increasing only from 0.764 to 0.767 (*Δ*AUC = 0.003), and this estimate was not internally validated. Accordingly, the findings should not be interpreted as demonstrating a clinically meaningful improvement in predictive performance.

Several mechanisms could explain the observed association, and the present data cannot distinguish among them. One possibility is that reduced perfusion pressure and ventilation-induced hypocapnia act through pressure-side and resistance-side mechanisms that converge on cerebral blood flow, particularly when autoregulation is impaired or shifted (1–3, 17). An equally plausible explanation is a common low-flow state: a fall in cardiac output or pulmonary blood flow can reduce both MAP and CO_2_ delivery to the lungs, thereby lowering EtCO_2_ independently of ventilation (18–20). Under this interpretation, DHCB may partly measure the same hemodynamic disturbance through two correlated signals. Low EtCO_2_ during hypotension could also function as a marker of the severity or individual physiological impact of hypotension, identifying patients with greater compromise related to cardiac function, volume status, ventilation–perfusion relationships, or autoregulatory reserve. Thus, the observed association does not establish that hypotension and hypocapnia are independent insults, nor does it demonstrate biological synergy or causal interaction. The term dual hit is an operational description of temporal concurrence; the primary inference is that the overlap contains outcome-related information beyond the corresponding non-overlapping exposure durations.

The interpretation of low EtCO_2_ in neurosurgical practice requires particular caution. Brief, deliberate hyperventilation may be requested to reduce cerebral blood volume, intracranial pressure, or brain bulk and improve operative conditions in selected settings (4, 5, 21). This scenario is clinically different from unplanned hypocapnia or hypocapnia that persists longer than necessary. Moreover, the need for aggressive brain relaxation may itself indicate edema, mass effect, reduced intracranial compliance, or technically difficult surgery, each of which may increase both hemodynamic instability and postoperative risk. The database did not contain the indication for hyperventilation, the timing of a surgeon's request, intracranial pressure, or a standardized brain-relaxation score; therefore, purposeful and inadvertent low EtCO_2_ could not be separated, and confounding by indication cannot be excluded. Our findings should not be interpreted as an argument against brief therapeutic hyperventilation. Rather, they identify temporal overlap with hypotension as a marker warranting prospective study in datasets that capture the reason for ventilation changes and the underlying intracranial condition.

The restricted cubic spline analysis demonstrated an overall association between DHCB and the composite outcome, but there was no statistically significant evidence of departure from linearity (*P* for nonlinearity = 0.143). Although the fitted curve showed local variation across selected DHCB values, these fluctuations should not be interpreted as evidence of a non-monotonic biological dose-response relation. The large proportion of operations with DHCB = 0 and sparse observations at higher burdens produced widening uncertainty and limit interpretation of the curve's shape. The spline and standardized-risk estimates therefore do not identify a clinical safety threshold.

The consistency of the association across progressively adjusted analyses provides some reassurance regarding robustness, but it does not eliminate the possibility of residual confounding or a shared low-flow mechanism. The effect estimate was only modestly attenuated after additional adjustment for preoperative laboratory measurements and diagnosis groups and remained positive after inclusion of blood loss, transfusion, vasopressor administration, median minute ventilation, and other ventilator settings. However, operation-level adjustment for minute ventilation cannot determine why EtCO_2_ was low at the exact time of hypotension, and the intraoperative-context model should not be regarded as the principal causal estimate. These intraoperative variables may reflect illness severity, clinical responses to deteriorating physiology, or processes occurring after exposure onset and may therefore act as mediators or colliders. The purpose of this model was to examine sensitivity to broader intraoperative context rather than to construct a maximally adjusted causal model. The similar estimate obtained when only the first eligible operation per patient was retained also suggests that the primary finding was not driven solely by repeated procedures within the same individuals.

The directionally consistent associations observed for prolonged postoperative hospitalization, prolonged ICU stay, and in-hospital mortality provide additional context, although these secondary analyses should be interpreted cautiously. The components differ substantially in clinical weight: death is an unequivocally severe event, whereas ICU duration and hospital length of stay are partly determined by institutional pathways, discharge practices, baseline function, and surgical complexity (13, 14, 22). They may also arise through different mechanisms and should not be assumed to represent a homogeneous biological construct. For this reason, the composite was used as an exploratory summary of adverse short-term recovery, and the individual components were reported separately.

Several limitations should be considered. First, the observational design precludes causal inference and cannot exclude residual confounding, reverse causation, or confounding by indication. Patients with more severe intracranial disease, greater brain swelling, mass effect, reduced intracranial compliance, technically difficult surgery, hemodynamic instability, or impaired neurological status may be more likely to receive intentional hyperventilation for brain relaxation, to become hypotensive, and to experience worse postoperative outcomes. Consistent with this concern, DHCB-positive operations more often involved open brain tissue excision and had longer anesthesia duration in the descriptive comparison. Restriction to open brain tissue excision produced a similar association, but this sensitivity analysis cannot account for within-procedure differences in edema, operative difficulty, or the indication for hyperventilation. Baseline neurological function, intracranial pressure, cerebral autoregulatory status, patient position, brain-relaxation assessment, and the clinical indication for ventilation changes could not be reliably reconstructed.

Second, EtCO_2_ was used as a surrogate for arterial carbon dioxide tension. The PaCO_2_–EtCO_2_ gradient may vary with ventilation–perfusion relationships, surgical position, and systemic hemodynamics. In particular, reduced cardiac output or pulmonary blood flow can lower CO_2_ delivery to the lungs and decrease EtCO_2_ independently of ventilation (18, 23). Consequently, concurrent hypotension and low EtCO_2_ may represent a common low-flow state rather than two independent physiological insults. Alternatively, low EtCO_2_ may be nested within the hypotension exposure as a marker of its severity or of greater individual physiological compromise related to cardiac function, volume status, autoregulation, or ventilation–perfusion mismatch. Although minute ventilation was available for most operations and was included in the intraoperative-context sensitivity model, operation-level ventilator summaries cannot resolve the contemporaneous mechanism. Cardiac index and stroke volume index were recorded in only 13.7% and 10.7% of operations, respectively, and their selective availability precluded reliable adjustment. The current observational design therefore cannot distinguish a pressure–vasoconstriction mechanism from a shared low-flow or severity-marker explanation, and DHCB should not be interpreted as proof of two independent causal hits.

Third, the publicly available INSPIRE dataset provides intraoperative vital signs at a maximum temporal resolution of 5 min; therefore, analyses at shorter intervals were not possible. Although this resolution enabled consistent temporal alignment of MAP and EtCO_2_, it may have obscured brief physiological events and cannot fully capture rapid transitions between exposure states. The estimated DHCB should therefore be interpreted as a time-weighted burden at the available temporal resolution rather than as a complete representation of second-to-second physiology. This approach enabled consistent temporal alignment of MAP and EtCO_2_ but may have missed brief physiological events and cannot fully capture rapid transitions between exposure states. The estimated DHCB should therefore be interpreted as a time-weighted burden at the available temporal resolution rather than as a complete representation of second-to-second physiology.

Fourth, procedure classification relied on truncated ICD-10-PCS information available in the public dataset. Although a high-specificity strategy was used to identify major open intracranial procedures, residual misclassification of procedure type and underlying pathology is possible. This limitation may also contribute to clinical heterogeneity across the analytic cohort.

Fifth, the exploratory composite primary outcome combined events of unequal clinical importance and potentially different mechanisms. In-hospital death carries substantially greater clinical weight than prolonged ICU stay or hospitalization, while the latter two are influenced by institutional care pathways and discharge practices. Neurologically specific outcomes such as new focal deficits, cerebral ischemia, delayed awakening, reoperation, postoperative mechanical ventilation, and validated neurological complications were not consistently available. Although individual components were reported separately and showed directionally consistent associations, the composite should be interpreted as a pragmatic marker of adverse short-term recovery rather than a homogeneous or neurologically specific endpoint.

Sixth, the incremental model-comparison analyses should be interpreted conservatively. Although addition of DHCB improved model fit, the increase in apparent AUC was only 0.003, and no bootstrap, cross-validation, or external validation was performed. These analyses support the presence of additional statistical information but do not establish clinically useful incremental predictive value.

Finally, this study used data from a single tertiary center in Korea. Case mix, thresholds for deliberate hyperventilation, blood pressure targets, arterial-line use, postoperative ICU admission, discharge practices, and neurocritical care pathways may differ across institutions and health systems. These setting-specific practices may affect both the distribution of DHCB and the measured outcomes, limiting transportability of the effect estimates. External validation in geographically and organizationally diverse multicenter cohorts is therefore required before DHCB can be considered for clinical risk stratification or intraoperative decision support.

Future research should prospectively specify the exposure, outcome, and covariate definitions before outcome analysis; incorporate neurologically specific postoperative endpoints; distinguish intentional therapeutic hyperventilation from unintentional low EtCO_2_ where possible; and evaluate arterial PaCO_2_ alongside EtCO_2_. Simultaneous measurement of cardiac output or stroke volume, minute ventilation, volume status, and cerebral autoregulation will be necessary to separate ventilation-mediated hypocapnia from low-flow reductions in EtCO_2_ and to determine whether DHCB represents two mechanistically distinct exposures or a marker of hypotension severity. Replication across different neurosurgical populations and institutions will be essential. More advanced causal analyses could also examine whether concurrent hypotension and hypocapnia interact on additive or multiplicative scales and whether reducing temporal overlap is associated with improved outcomes. Only after such validation should intervention thresholds or real-time monitoring strategies be considered.

## Conclusion

Among adults undergoing major open intracranial surgery, greater temporal overlap between intraoperative hypotension and low EtCO_2_ was associated with adverse short-term postoperative outcomes beyond the corresponding non-overlapping exposure burdens. Although DHCB improved model fit, its incremental discrimination was small, and no significant departure from linearity or clear safety threshold was identified. DHCB should be interpreted as a hypothesis-generating marker of concurrent physiological exposure, not as evidence that two independent causal insults or biological synergy have been established. External validation using neurologically specific outcomes and contemporaneous measures of ventilation and cardiac output is required before clinical application.

## Data Availability

Publicly available datasets were analyzed in this study. This data can be found here: https://doi.org/10.13026/1eay-yc85
