# Supplementary material for: Temporal overlap of intraoperative hypotension and hypocapnia and postoperative outcomes after major open intracranial surgery: a time-weighted dual-hit cohort study
Source: Front Med (Lausanne). 2026 Oct 1;13:1946283. doi: 10.3389/fmed.2026.1946283 (PMC13669116; doi:10.3389/fmed.2026.1946283)
Supplement: Supplementary file 1 [file Supplementaryfile1.docx]

Supplement

[**Table S1. Characteristics of operations according to the presence or absence of concurrent hypotension and hypocapnia.**	2](#_Toc235695325)

[**Table S2. Incremental information after adding DHCB.**	4](#_Toc235695326)

[**Table S3. Patient-clustered associations between DHCB and the composite adverse outcome across adjustment strategies.**	5](#_Toc235695327)

[**Table S4. Marginally standardized absolute risks and risk differences.**	6](#_Toc235695328)

[**Table S5. Patient-clustered sensitivity analyses for the composite adverse outcome.**	7](#_Toc235695329)

# **Table S1. Characteristics of operations according to the presence or absence of concurrent hypotension and hypocapnia.**

| **Characteristic** | **DHCB=0 (n=1578)** | **DHCB>0 (n=708)** |
| --- | --- | --- |
| Age, years | 55.0 [45.0–65.0] | 60.0 [45.0–65.0] |
| Female sex | 889 (56.3%) | 410 (57.9%) |
| BMI, kg/m² | 23.9 [21.5–26.0] | 22.9 [20.8–25.7] |
| ASA physical status I–II | 1,332 (84.4%) | 570 (80.5%) |
| ASA physical status III | 173 (11.0%) | 96 (13.6%) |
| ASA physical status IV–V | 18 (1.1%) | 10 (1.4%) |
| ASA physical status, missing | 55 (3.5%) | 32 (4.5%) |
| Emergency surgery | 159 (10.1%) | 79 (11.2%) |
| Open brain tissue excision | 1,020 (64.6%) | 553 (78.1%) |
| Open intracranial vascular surgery | 382 (24.2%) | 83 (11.7%) |
| Open intracranial drainage | 176 (11.2%) | 72 (10.2%) |
| Anesthesia duration, min | 255 [145–345] | 305 [220–400] |
| Preoperatively recorded hypertension | 99 (6.3%) | 44 (6.2%) |
| Preoperatively recorded diabetes mellitus | 67 (4.2%) | 44 (6.2%) |
| Preoperatively recorded chronic kidney disease | 13 (0.8%) | 9 (1.3%) |
| Preoperatively recorded ischemic heart disease | 39 (2.5%) | 20 (2.8%) |
| Preoperatively recorded cerebrovascular disease | 179 (11.3%) | 77 (10.9%) |
| Preoperatively recorded malignancy | 493 (31.2%) | 249 (35.2%) |
| Hemoglobin, g/dL | 13.2 [12.5–14.2] | 13.2 [12.2–14.2] |
| Creatinine, mg/dL | 0.74 [0.65–0.88] | 0.74 [0.65–0.88] |
| Albumin, g/dL | 4.1 [4.0–4.4] | 4.1 [3.9–4.3] |
| Sodium, mmol/L | 140 [139–141] | 139 [137–141] |
| Glucose, mg/dL | 120 [101–138] | 120 [101–138] |
| Platelet count, ×10⁹/L | 233 [200–273] | 233 [200–291] |
| White blood cell count, ×10⁹/L | 6.67 [5.58–8.90] | 7.05 [5.58–8.90] |
| Hypotension duration, min | 10 [0–35] | 40 [20–95] |
| Hypocapnia duration, min | 5 [0–25] | 45 [15–130] |
| DHCB duration, min | 0 [0–0] | 10 [5–25] |
| DHCB proportion, % | 0.0 [0.0–0.0] | 3.9 [1.9–8.9] |
| Composite adverse outcome | 398 (25.2%) | 278 (39.3%) |
| Prolonged postoperative hospitalization | 337 (21.4%) | 235 (33.2%) |
| ICU stay ≥2 days | 157 (9.9%) | 119 (16.8%) |
| In-hospital death | 25 (1.6%) | 28 (4.0%) |

Continuous variables are median [IQR]; categorical variables are n (%). Diagnosis groups were derived from codes recorded before anesthesia and may underestimate chronic disease prevalence. This descriptive comparison is not intended for causal inference.

# **Table S2. Incremental information after adding DHCB.**

| **Model** | **Log likelihood** | **AIC** | **BIC** | **Apparent AUC** | **ΔAUC** | **Cluster-robust Wald χ²** | **P value** |
| --- | --- | --- | --- | --- | --- | --- | --- |
| Model A: clinical covariates + hypotension-only + hypocapnia-only | -1154.43 | 2336.86 | 2417.15 | 0.764 | Reference | – | – |
| Model B: Model A + DHCB | -1145.76 | 2321.53 | 2407.55 | 0.767 | +0.003 | 19.36 | <0.001 |

Model A included the prespecified clinical covariates, hypotension-only burden, and hypocapnia-only burden. Model B additionally included DHCB. The Wald test used patient-clustered robust covariance. AIC, BIC, and AUC are descriptive in-sample measures; no resampling-based internal validation was performed.

# **Table S3. Patient-clustered associations between DHCB and the composite adverse outcome across adjustment strategies.**

| **Analysis** | **Operations** | **Patients** | **aOR** | **95% CI** | **P value** |
| --- | --- | --- | --- | --- | --- |
| Core clinical model | 2,286 | 2,092 | 1.19 | 1.10–1.28 | <0.001 |
| Preoperative laboratories and diagnoses | 2,286 | 2,092 | 1.17 | 1.08–1.26 | <0.001 |
| Intraoperative context | 2,286 | 2,092 | 1.15 | 1.06–1.25 | <0.001 |
| First eligible operation per patient | 2,092 | 2,092 | 1.20 | 1.11–1.29 | <0.001 |

Effect estimates are reported per 5-percentage-point increase in DHCB. The first three models used patient-level cluster-robust standard errors; the first-operation analysis retained one operation per patient. The intraoperative-context model additionally included estimated blood loss, transfusion, vasopressor use, median minute ventilation, tidal volume, positive end-expiratory pressure, peak inspiratory pressure, and inspired oxygen fraction. Cardiac index and stroke volume index were not included because they were available in only 313 (13.7%) and 244 (10.7%) operations, respectively.

# **Table S4. Marginally standardized absolute risks and risk differences.**

| **DHCB** | **Adjusted risk** | **95% CI** | **Risk difference, percentage points** | **95% CI** |
| --- | --- | --- | --- | --- |
| 0% | 27.3% | 25.2–29.3% | Reference | – |
| 5% | 34.7% | 30.0–39.4% | +7.4 | 2.1 to 12.8 |
| 10% | 33.4% | 27.7–39.1% | +6.1 | -0.02 to 12.3 |
| 20% | 37.1% | 29.2–45.0% | +9.9 | 1.7 to 18.0 |
| 30% | 45.6% | 36.9–54.4% | +18.4 | 9.4 to 27.4 |

Risks were estimated from the restricted cubic spline model and standardized over the analytic cohort. Confidence intervals used the delta method with patient-clustered covariance. Estimates at higher DHCB levels were supported by fewer observations.

# **Table S5. Patient-clustered sensitivity analyses for the composite adverse outcome.**

| **Analysis** | **Operations** | **aOR** | **95% CI** | **P value** |
| --- | --- | --- | --- | --- |
| Primary analysis | 2,286 | 1.19 | 1.10–1.28 | <0.001 |
| Elective surgery only | 2,048 | 1.19 | 1.10–1.29 | <0.001 |
| Open brain tissue excision only | 1,573 | 1.19 | 1.09–1.29 | <0.001 |
| First operation per patient | 2,092 | 1.20 | 1.11–1.29 | <0.001 |
| Invasive MAP coverage >=80% | 2,000 | 1.16 | 1.08–1.25 | <0.001 |
| MAP <70 and EtCO2 <30 mmHg | 2,286 | 1.12 | 1.07–1.18 | <0.001 |
| MAP <65 and EtCO2 <32 mmHg | 2,286 | 1.04 | 1.01–1.08 | 0.023 |

Effect estimates are reported per 5-percentage-point increase in the threshold-specific concurrent burden. For each alternative threshold definition, hypotension-only, hypocapnia-only, and concurrent burdens were reconstructed as mutually exclusive states and entered simultaneously. Patient-level cluster-robust standard errors were used whenever multiple operations per patient were retained.
